# Improving the Quality of Old Age Inpatient Ward Rounds During COVID-19 in NHS Lanarkshire, Scotland

**DOI:** 10.1192/bjo.2022.267

**Published:** 2022-06-20

**Authors:** Prerna Ambardar, Indrajit Chatterji

**Affiliations:** NHS, Lanarkshire, Glasgow, United Kingdom

## Abstract

**Aims:**

To provide a structured Multidisciplinary team (MDT) checklist to improve the quality of ward rounds in Ward 3, Wishaw General hospital. Ward rounds normally involved patient and electronic documentation review during MDT. Feedback from medical and nursing staff indicated inconsistencies in finding up to date Do not resuscitate cardiopulmonary resuscitation (DNACPR)forms/Treatment escalation plans, treatment forms, care plans, thromboembolism prophylaxis during ward rounds. Discussions about who will update the family, make social work referrals needed clear documentation in order to follow up efficiently post MDT. Food, fluid, and weight charts although done regularly there was no single place to keep them together. These charts to be kept in a separate folder for finding easily during ward rounds. The Royal College of Psychiatrists sets standards that managers and practitioners have agreed standards for ward rounds. Structured ward rounds and check lists have shown to prevent omissions in care and improve patient safety.

**Methods:**

Discussions with Ward 3 team, nursing colleagues and ward Quality improvement group were held. A Standard MDT Quality improvement Checklist was devised and used as a Pilot in W3, WGH.

This was first introduced in August 2021. Plan, do, study, act (PDSA) cycle was carried out.

Plan: Trial MDT checklist at Ward 3 ward Round

Do: Use Initially for two Consultant ward rounds

Study: Ask all MDT staff members for feedback on the form

Act: Reformat the checklist for the following ward rounds and distribute among all consultants.

Repeated Revisions of MDT checklist done after feedback from ward staff and final version devised and results audited in Nov 2021 and Jan 2022.

Food, Fluid, and weight charts were put in separate folders.

**Results:**

Before MDT Checklist nil up to date MDT checklist information available, 10% individual food and fluid charts and 0% folders.

After MDT checklist in November 2021, 73% increase in up-to-date checklist items, 100% increase in finding charts in folders.

In January 2022 decrease to 44% of up-to-date MDT checklist items, 100% food and fluid charts in folders.

**Conclusion:**

MDT Checklist provided robust structure to our Ward rounds along with the regular electronic record and has been incorporated in our shared drive. The results in January for up-to-date checklist were down because of staff sickness due to new Omicron variant and less people available to keep documentation up-to-date.

